# Syphilitic Manifestations in the Mouth and Pharynx

**Published:** 1890-02

**Authors:** Frank O. Stockton

**Affiliations:** Atlanta, Ga.; Late Professor of Laryngology in College of Physicians and Surgeons, and Chicago Polyclinic, Chicago, Illinois


### SYPHILITIC MANIFESTATIONS IN THE MOUTH AND PHARYNX.

BY FRANK O. STOCKTON, M. D., ATLANTA, GA.

Late Professor of Laryngology in College of Physicians and Surgeons, and Chicago Polyclinic, Chicago, Illinois.

Syphilitic manifestations in the throat are usually of a secondary nature, due to the general systemic poison. Occasionally, however, it is the result of direct inoculation from the primary chancre by actual contact.

The initial sore has been seen on the tonsils, palatine folds, and even on the posterior wall of the pharynx, and in one instance, on the laryngeal surface of the epylottis. Syphilis is sometimes communicated by kissing. It has been known to follow the drawing of the nipple by the mouth of a syphilitic nurse. Children frequently contract it from the nipple of a syphilitic wet nurse. It is also contracted by the use of implements belonging to syphilitic persons, such as pipes, glasses, etc., and frequently through the use of surgical instruments.

In case of chancre of the tongue, that organ frequently becomes the seat of a phagadenic ulceration, which is apt to be mistaken for epithelioma or furuncle, and possibly tuberculous or cancerous ulcerations.

These cases, as a rule, are not seen in their early stages, owing to the shame of the patient, who delays going to the physician until absolutely necessary.

The manifestations of syphilis that we encounter in the pharynx are, specific catarrhal pharyngitis, mucus patches, or condolomita, superficial and deep ulcers, and occasionally a gummy tumor.

*Specific Laryngitis*, or ordinary syphilitic sore throat, may occur as early as three weeks after the primary sore, or it may not appear for months. Its characteristics are those of ordinary sore throat, so that a diagnosis of its specific character is sometimes impossible without the history. Usually the line of demarcation is very distinct, while in the non-specific the diseased tissue gradually shades off into the healthy. In case there is any eruption on the skin, or other secondary symptoms of syphilis, the diagnosis is easy.

The treatment is the same as for an ordinary sore throat with the addition of the constitutional treatment for syphilis.

The bichloride of mercury should be given three times daily in 1-16 of a grain doses, and kept up for a year or more, then the doses may be gradually reduced.

*Mucus Patches* are usually considered a secondary manifestation of syphilis, but as has been truly said of them, they are the first to come and the last to go of syphilitic symptoms. It is doubtful which stage they belong to.

These patches consist of an infiltration into the superficial layer of the mucous membrane of lymph cells, which gives them the appearance of tissue touched with the nitrate of silver, when seen in their early stages. Soon they extend laterally and in exposed positions ulcerate. Their favorite place in the throat is on the tonsil, next on the pillars of the fauces, extending to the soft palate and uvula, frequently forming a border to the last organs. It is also found on the sides of the tongue and inner surface of the cheeks—rarely on the posterior wall of the pharynx.

These patches are the most infectious of the secondary lesions.

A notable character of these patches is their symmetry. When a patch is confined to the tonsil, a nice discrimination is required to determine its character, as a hypertrophied tonsil will frequently present on its surface the greyish and mottled appearance of a mucus patch. The resemblance to the appearance produced by caustics must also be borne in mind.

A prominent symptom of the mucus patch is pain, particularly if seated in the fauces; the pain is of a sharp and pricking character.

Most anthorities recommend that these patches be destroyed by caustic. I have found it a good plan to scrape off the patch and coat the denuded surface with iodoform.

The infectious nature of these patches must be remembered in treating them, as the necessary maniipulation of the parts is apt to cause a sudden cough, which is liable to throw some of the secretion into the eye, or some other vulnerable part, as several recorded instances show where doctors have lost their sight through chancre of the cornea; also contracted constitutional syphilis.

*Superficial Ulcerations* occur as early as the third month, or as late as four years after the primary lesion. This is a moderately active destructive process, confined to the mucous membrane.

It usually begins as a simple erosion, but occasionally commences in a mucus patch that has been subjected to irritation.

Its fovorite location is on the lateral wall of the pharynx, near the posterior pillars, extending to the pillars and tonsil frequently. It is rarely seen on the soft palati and uvula.

The ulcerations are generally of a rounded outline, with a greyish-yellow surface, slightly depressed below the surrounding mucous membrane. The edges are sharp-cut, and the areola moderately well marked.

The secretions are limited in quantity, and of a purulent character.

These ulcerations are not generally painful, except in swallowing.

In these cases, in addition to the general treatment, the local trouble must be faithfully watched and treated, as the ulceration process is apt to lead to loss of tissue in some parts, which might result in a serious impairment of their functions, as in case of the soft palate and the pillars of the fauces.

It is generally recommended to destroy these ulcers with some destructive agent, such as the actual and galvano-cautery nitrate of silver, etc., all of which are extremely painful, and also of necessity causes an additional loss of substance, which it seems to me is radically wrong, inasmuch as we have a remedy which is so nearly specific in its action that nothing more could be desired, namely, iodoform.

It may be applied pure or combined with morphia, or, in fact, most any substance. The surface should be carefully cleaned before applying the drug.

*Deep or Tertiary* ulcerations extend, as a rule, over a period from four to twenty years after the primary sore, but occur usually between the seventh and tenth year. The character of these last ulcerations are entirely different from those I have previously attempted to describe. They are extremely virulent incharacter, and rapidly destructive, both laterally and deeply. Very often grave and serious injuries are caused merely from the amount of tissue destroyed. Another peculiarity is the very great contractions which result from the cicatrices.

These ulcerations are in most, if not all, cases caused by the breaking down of gummy tumors deposited in the deep layer of the mucous membranes.

The destructive process follows so soon after the deposit of the gummy materials that it is extremely rare to see a case before the ulcerations have begun.

The gummy deposit occurs in the sub-mucous layers of the mucous membrane in the form of small rounded nodules, either singly or in groups—generally they form extensive groups. This new material begins to soften and break down in the centre, and extends outwards, and in this way reaches the surface, giving rise to the tertiary ulcers.

These ulcers have sharp-cut, jagged, overhanging edges; the surface of the ulcer is depressed and excavated, and covered with a grayish-yellow purulent discharge, and sometimes presents the appearance of gangrene.

The surrounding tissue is characteristic and peculiar. It is actively and acutely inflamed, greatly congested and swollen producing a discoloration unlike any other form of inflammation, and once seen never to be forgotten. It might be described as a coppery purplish red.

The favored site of these ulcers, in order, is the tonsil, soft-palate, and uvula, and the posterior wall of the pharynx Wherever they are located, their tendency is to extend to the surrounding parts.

The cicatrices resulting from these deep ulcers, as was before stated, are marked by extensive contractions, which frequently result in great deformity.

These deformities are due partly to the cicatrization and partly to the abnormal adhesions, and also to the loss of tissue. The most frequent deformity is met with in the soft-palate caused by the loss of substance and to being drawn to one side by the abnormal contractions and adhesions. Frequently the soft-palate and the posterior wall of the pharynx are joined by the adhesions, so that the normal openings are completely closed.

*Treatment.*—The early recognition of these ulcers and their prompt and vigorous treatment is of the greatest importance, as their progress is so rapid that they result in a serious loss of tissue in a very short time.

This is the time when the iodide of potass. is of the greatest use. I usually begin with 15 grains three times daily, and increase the amount 3 grains daily until I see a decided change in the character of the ulcers, or till iodinism is produced. Mercury seems to be of very little use, and I am rather inclined to think it should not be given at all while the ulcerative action lasts.

Local treatment consists in thoroughly cleaning the parts of the products of ulceration, and then applying iodoform, either pure or mixed with morphine, and starch over the whole diseased surface.

The deformities which the cicatrices produce are frequently the source of great distress, particularly those which cause the complete closure of the nase-pharyngeal space.

Many operations have been tried for these deformities, but none of them are very successful, or, at least, only successful so long as the patient is under treatment. So, it appears that the most we can do is to palliate the difficulty.

